# Supplementary material for: Use of Digital Tools for the Assessment of Food Consumption in Brazil: A Scoping Review
Source: Nutrients. 2024 May 6;16(9):1399. doi: 10.3390/nu16091399 (PMC11085537; doi:10.3390/nu16091399)
Supplement: Supplementary file 1 [file nutrients-16-01399-s001.zip › nutrients-2957803-supplementary.pdf]

Table S1. Summary of characteristics for all studies (n = 94) included in the scoping review.

Articles					Population				Type of tool			Type of technology	
**Study number**	**Author(Reference)**	**Year**	**Type of Study**	**Food Consumption Assessment Methods**	**Children**	**Adolescents**	**Adults**	**Older adults**	**PC / Laptop**	**Smartphone/ Tablet**	**Palm/ PDA**	**Web-based**	**Application**
1	Alcantara et al.[25]	2021	cross-sectional	Food Frequency Questionnaire			x		x	x		x	
2	Alves et al.[26]	2019	cross-sectional	24-hour recall		x					x		x
3	Azarias[27]	2020	validation	Food Frequency Questionnaire			x		x	x		x	
4	Baldo et al. [28]	2015	tool development	Food consumption markers			x	x		x			x
5	Barufaldi et al.[29]	2016	cross-sectional	24-hour recall		x					x		x
6	Bel-Serrat et al.[30]	2017	tool development	24-hour recall			x	x	x				x
7	Boing et al.[31]	2014	cross-sectional	24-hour recall			x				x		x
8	Borges[32]	2019	tool development	Food record			x			x		x	
9	Botelho[33]	2021	cross-sectional	24-hour recall			x		x	x		x	
10	Brasil, VIGITEL[34]	2006	cross-sectional	Food consumption markers			x	x	x				x
11	Brasil, VIGITEL[35]	2007	cross-sectional	Food consumption markers			x	x	x				x
12	Brasil, VIGITEL[36]	2008	cross-sectional	Food consumption markers			x	x	x				x
13	Brasil, VIGITEL[37]	2009	cross-sectional	Food consumption markers			x	x	x				x
14	Brasil, VIGITEL[38]	2010	cross-sectional	Food consumption markers			x	x	x				x

Table S1. *Cont.*

Articles					Population				Type of tool			Type of technology	
Study number	Author(Reference)	Year	Type of Study	Food Consumption Asssessment Methods	Children	Adolescents	Adults	Older adults	PC / Laptop	Smartphone/ Tablet	Palm/ PDA	Web-based	Application
15	Brasil, VIGITEL[39]	2011	cross-sectional	Food consumption markers			x	x	x				x
16	Brasil, VIGITEL[40]	2012	cross-sectional	Food consumption markers			x	x	x				x
17	Brasil, VIGITEL[41]	2013	cross-sectional	Food consumption markers			x	x	x				x
18	Brasil, VIGITEL[42]	2014	cross-sectional	Food consumption markers			x	x	x				x
19	Brasil, VIGITEL[43]	2015	cross-sectional	Food consumption markers			x	x	x				x
20	Brasil, VIGITEL[44]	2016	cross-sectional	Food consumption markers			x	x	x				x
21	Brasil, VIGITEL[45]	2017	cross-sectional	Food consumption markers			x	x	x				x
22	Brasil, VIGITEL[46]	2018	cross-sectional	Food consumption markers			x	x	x				x
23	Brasil, VIGITEL[47]	2019	cross-sectional	Food consumption markers			x	x	x				x
24	Brasil, VIGITEL[48]	2020	cross-sectional	Food consumption markers			x	x	x				x
25	Brasil, VIGITEL[49]	2021	cross-sectional	Food consumption markers			x	x	x				x
26	Caivano Domene[51]	2013	cross-sectional	24-hour recall			x			x			x
27	Caivano Ferreira Domene[52]	2014	cohort	Diet Quality Index			x			x			x
28	Caivano; Colugnati Domene[50]	2019	Cross-sectional	Digital Food Guide			x		x				x

Table S1. *Cont.*

Articles					Population				Type of tool			Type of technology	
Study number	Author(Reference)	Year	Type of Study	Food Consumption Asssessment Methods	Children	Adolescents	Adults	Older adults	PC / Laptop	Smartphone/ Tablet	Palm/ PDA	Web-based	Application
29	Carvalho-Ferreira et al.[54]	2018	Cross-sectional	Food Preference Questionnaire			x		x	x		x	
30	Claro et al.[53]	2015	cross-sectional	Food Frequency Questionnaire			x				x	x	
31	Costa et al.[57]	2013	tool development	Food record	x	x			x			x	
32	Costa et al.[55]	2021	Cross-sectional	Food consumption markers			x			x			x
33	Costa et al.[56]	2023	cross-sectional	24-hour recall			x		x	x		x	x
34	Da Costa et al.[58]	2013	tool development	Food record	x	x			x			x	
35	Davies et al.[59]	2014	validation	Food record	x	x			x			x	
36	Dias[60]	2017	cross-sectional	Food Frequency Questionnaire			x		x			x	
37	Ducci[61]	2010	cross-sectional	24-hour recall	x				x			x	
38	Duque[62]	2020	tool development	Food record					x	x		x	
39	Engel et al.[63]	2017	validation	Food items	x				x			x	
40	Ferreira[64]	2017	cross-sectional	Food Frequency Questionnaire		x				x		x	
41	Folchetti et al.[65]	2015	tool development	Food Frequency Questionnaire			x		x	x		x	
42	Folchetti[66]	2016	tool development	Food Frequency Questionnaire			x		x	x		x	

Table S1. ***Cont.***

Articles					Population				Type of tool			Type of technology	
Study number	Author[(Reference)]	Year	Type of Study	Food Consumption Assessment Methods	Children	Adolescents	Adults	Older adults	PC / Laptop	Smartphone/ Tablet	Palm/ PDA	Web-based	Application
43	Galante[67]	2004	Cross-sectional	Food Frequency Questionnaire			x			x		x	
44	Galante[68]	2007	validation	Food record			x			x		x	
45	Galante[69]	2008	tool development	Food Frequency Questionnaire			x			x		x	
46	Hinnig[70]	2014	Cross-sectional	Food Frequency Questionnaire	x				x				x
47	IBGE - PeNSE, 2009[71]	2009	cross-sectional	Food Frequency Questionnaire		x					x		x
48	IBGE - PeNSE, 2012[72]	2013	cross-sectional	Food Frequency Questionnaire		x					x		x
49	IBGE - PeNSE, 2015[73]	2016	cross-sectional	Food Frequency Questionnaire		x					x		x
50	IBGE - PeNSE, 2019[74]	2021	cross-sectional	Food Frequency Questionnaire		x					x		x
51	IBGE - PNS, 2013[75]	2015	cross-sectional	Food Frequency Questionnaire			x	x			x		x
52	IBGE - PNS, 2019[76]	2021	cross-sectional	Food Frequency Questionnaire			x	x		x			x
53	IBGE - PNAD, 2015[77]	2016	cross-sectional	Brazilian Scale for Food Insecurity - EBIA			x	x	x	x			x
54	IBGE - POF 2017-2018[78]	2020	cross-sectional	24-hour recall		x	x	x		x			x
55	Jesus Assis Kupek[79]	2017	validation	Food items	x	x			x			x	
56	Kac G[80]	2020	cohort	24-hour recall	x					x			x

Table S1. *Cont.*

Articles					Population				Type of tool			Type of technology	
Study number	Author[(Reference)]	Year	Type of Study	Food Consumption Assessment Methods	Children	Adolescents	Adults	Older adults	PC / Laptop	Smartphone/ Tablet	Palm/ PDA	Web-based	Application
57	Karnopp[81]	2016	cross-sectional	Food Frequency Questionnaire		x			x	x		x	
58	Lacerda et al.[82]	2021	tool development	24-hour recall	x					x			x
59	Lobo et al.[83]	2019	cross-sectional	24-hour recall	x	x			x			x	
60	Maciel[84]	2022	cross-sectional	Food items			x			x		x	
61	Medeiros; Mainbourg[85]	2023	cross-sectional	24-hour recall			x	x		x		x	
62	Maia et al.[86]	2018	cross-sectional	Food consumption markers		x				x			x
63	Malta et al.[87]	2021	cross-sectional	Food consumption markers			x		x				x
64	Maynard et al.[88]	2020	cross-sectional	Food Frequency Questionnaire			x			x		x	
65	Meneghini Netto[89]	2021	cross-sectional	24-hour recall		x				x		x	
66	Mescoloto[90]	2018	Cross-sectional	24-hour recall			x			x			x
67	Mescoloto Caivano Domene[91]	2017	cross-sectional	24-hour recall			x			x			x
68	Molina et al.[92]	2013	tool development	Food Frequency Questionnaire			x	x	x				x
69	Neri et al.[93]	2023	cohort	24-hour recall			x		x	x		x	
70	Neves[94]	2019	cross-sectional	24-hour recall		x			x				x

Table S1. *Cont.*

Articles					Population				Type of tool			Type of technology	
**Study number**	**Author(Reference)**	**Year**	**Type of Study**	**Food Consumption Assessment Methods**	**Children**	**Adolescents**	**Adults**	**Older adults**	**PC / Laptop**	**Smartphone/ Tablet**	**Palm/ PDA**	**Web-based**	**Application**
71	Ochsenhofer[95]	2007	validation	Food Frequency Questionnaire			x		x	x		x	
72	Oliveira et al.[96]	2016	cross-sectional	24-hour recall		x			x			x	
73	Oliveira et al.[97]	2022	cross-sectional	24-hour recall			x	x	x			x	
74	Oliveira et al.[98]	2023	validation	Food items	x				x	x			
75	Ramos[99]	2013	cross-sectional	Food Frequency Questionnaire		x			x			x	
76	Rede Penssan[100]	2021	cross-sectional	Food consumption markers	x	x	x	x		x		x	
77	Rede Penssan[101]	2022	cross-sectional	Food consumption markers	x	x	x	x		x		x	
78	Retondario et al.[102]	2019	cross-sectional	24-hour recall		x			x			x	
79	Ruggeri et al.[103]	2012	tool development	24-hour recall	x	x			x			x	x
80	Ruggeri et al.[104]	2013	validation	24-hour recall	x	x			x			x	x
81	Ruiz-Roso et al.[105]	2020	cross-sectional	Food consumption markers		x			x			x	
82	Santos et al.[106]	2019	tool development	Food record			x			x			x
83	Sattamini[107]	2019	validation	Food Frequency Questionnaire			x			x			x
84	Schneider et al.[8]	2016	tool development	Food Frequency Questionnaire		x	x			x		x	

Table S1. *Cont.*

Articles					Population				Type of tool			Type of technology	
**Study number**	**Author(Reference)**	**Year**	**Type of Study**	**Food Consumption Assessment Methods**	**Children**	**Adolescents**	**Adults**	**Older adults**	**PC / Laptop**	**Smartphone/ Tablet**	**Palm/ PDA**	**Web-based**	**Application**
85	Silva et al.[108]	2016	cross-sectional	24-hour recall		x			x			x	
86	Silva et al.[109]	2021	cross-sectional	24-hour recall				x	x				x
87	Silva et al.[110]	2021	cross-sectional	Food consumption markers			x		x	x		x	
88	Silva et al.[111]	2021	tool development	Food Frequency Questionnaire			x			x		x	
89	Souza et al.[112]	2016	cross-sectional	24-hour recall		x			x			x	
90	Steele et al.[113]	2020	cohort	24-hour recall			x		x	x		x	
91	Steluti et al.[9]	2020	tool development	24-hour recall			x		x				x
92	Voci[114]	2011	cross-sectional	Food Frequency Questionnaire		x			x				x
93	Yamashita et al.[115]	2019	validation	Food Frequency Questionnaire			x		x			x	
94	Zanchim Kirten Marchi[116]	2018	cross-sectional	Food consumption markers			x	x		x			x

Table S2. Summary of characteristics for digital tools (n = 48) included in the scoping review.

Digital tool number	Digital tool	Author[(Reference)]	Year	Type of Study	Aims	Time (minutes)	Free Access
**1**	Online questionnaire - Google Forms	Alcantara et al.[25]	2021	cross-sectional	To evaluate the use of Food Delivery Applications (FDAs) and their influence on the eating habits of the population of the greater São Paulo		
**2**	CUME - Online questionnaire - The Cohort of Universities of Minas Gerais	Azarias[27]	2020	validation	To describe the construction of the online Food Frequency Questionnaire (FFQ) used at CUME (Cohort of Universities of Minas Gerais, Brazil) and evaluate its validity and reproducibility		X
**3**	Diabetes Food Control App	Baldo et al.[28]	2015	tool development	To present the application Diabetes Food Control, developed to evaluate the diabetic's feeding, based on a validated questionnaire.		X
		Zanchim, Kirten, Marchi[116]	2018	cross-sectional	To assess the consumption of dietary intake markers in patients with diabetes using a mobile application.		x
**4**	ERICA – REC24HR - Study of Cardiovascular Risk Factors in Adolescents	Alves et al.[26]	2019	cross-sectional	To identify the dietary patterns of Brazilian adolescents in each of Brazil's five major geographic regions and verify possible differences in adherence to dietary patterns according to age, sex, and type of school	20 minutes	X
		Barufaldi et al.[29]	2016	cross-sectional	To describe the development of a tool for data collection of 24-hour food recall (REC24h) in ERICA and to report its performance in the pilot study	20 minutes	X
		Neves[94]	2019	cross-sectional	To describe the consumption of sugary drinks and their association with cardiovascular risk factors in Brazilian adolescents.	20 minutes	X
		Oliveira et al.[97]	2016	cross-sectional	To describe the length of exposure to screens and the prevalence of consumption of meals and snacks by Brazilian adolescents in front of screens.	20 minutes	X
		Retondario et al.[102]	2019	cross-sectional	To assess usual intake of selenium (Se) and dietary inadequacies regarding the consumption of this mineral, and to identify the main dietary sources of Se among 12–17-year-old Brazilian adolescents.	20 minutes	X
		Silva et al.[108]	2016	cross-sectional	To describe the response percentage and characteristics of participants and non-participants in the Study of Cardiovascular Risks in Adolescents (ERICA) according to subsets of information.	20 minutes	X

Table S2. *Cont.*

Digital tool number	Digital tool	Author(Reference)	Year	Type of Study	Aims	Time (minutes)	Free Access
**4**	ERICA – REC24HR - Study of Cardiovascular Risk Factors in Adolescents	Bel-Serrat et al.[30]	2017	tool development	The present study describes the procedure and approaches needed to adapt and harmonise the GloboDiet methodology, a computer- and interview-based 24 h dietary recall, for use in two Latin American pilot countries, Brazil and Mexico.	20 minutes	X
**5**	Online questionnaire in Personal Digital Assistant (PDA)	Boing et al.[31]	2014	cross-sectional	The present study aims at describing the sampling plan, operational aspects and strategies used to optimize the field work of a cross-sectional, population-based study conducted in a southern capital of Brazil.	50 minutos	Yes
**6**	Photographed food records	Borges[32]	2019	tool development	To develop and validate a protocol for performing food records photographed by visually impaired people using a cell phone camera.		
**7**	Online survey questionnaire using the Google Forms platform	Botelho[33]	2021	cross-sectional	To describe ready-to-eat food delivery apps users and the characteristics of the use profile according to the frequency of use of such platforms, before and during physical distancing due to the COVID-19 pandemic		
**8**	Electronic Questionnaire - Vigitel	Brasil, VIGITEL[34-49]	2006<br>2007<br>2008<br>2009<br>2010<br>2011<br>2012<br>2013<br>2014<br>2015<br>2016<br>2017<br>2018	cross-sectional	To monitor the frequency and distribution of risk and protective factors for chronic non-communicable diseases in all capitals of the 26 Brazilian states and the Federal District.		

Table S2. *Cont.*

Digital tool number	Digital tool	Author(Reference)	Year	Type of Study	Aims	Time (minutes)	Free Access
8	Electronic Questionnaire - Vigitel	Brasil, VIGITEL [34-49]	2006<br>2020<br>2021	cross-sectional	To monitor the frequency and distribution of risk and protective factors for chronic non-communicable diseases in all capitals of the 26 Brazilian states and the Federal District.		
		Malta et al.[87]	2021	cross-sectional	To analyze the temporal trend of the prevalence of alcohol abuse among adults in Brazilian capitals, between 2006 and 2019.		
9	The Diet Quality Index associated with the Digital Food Guide (DQI-DFG	Caivano; Colugnati, Domene50	2019	cross-sectional	To improve and validate the DQI-DFG for a second version		X
		Caivano, Domene[51]	2013		To present a Diet Quality Index proper for dietary intake studies of Brazilian adults.		X
		Caivano, Ferreira, Domene[52]	2014	cohort	To evaluate user perception regarding the usability of the Digital Food Guide (DFG), which is a mobile smartphone app with guidelines on healthy eating.		X
10	Online Questionnaire - Pesquisa Nacional de Saúde (PNS) / National Health Survey (NHS)	Claro, et al.[53]	2015	cross-sectional	To describe the consumption of unhealthy foods considered risk factors for chronic non-communicable diseases (NCDs) according to regional and sociodemographic characteristics of Brazilian adults		
		IBGE - PNS, 2013[75]	2015		To investigate various socioeconomic and demographic characteristics that are included in the system according to information needs.		
		IBGE - PNS, 2019[76]	2021		To collect information on the performance of the national health system with regard to access and use of available services and continuity of care, as well as on the health conditions of the population, surveillance of chronic non-communicable diseases and risk factors them associated.		
11	Leeds Food Preference Questionnaire (LFPQ)	Carvalho-Ferreira et al.54	2018	cross-sectional	To translate and adapt the Leeds Food Preference Questionnaire (LFPQ) for the Brazilian population.		

Table S2. *Cont.*

Digital tool number	Digital tool	Author(Reference)	Year	Type of Study	Aims	Time (minutes)	Free Access
**12**	Nova screener for the consumption of ultra-processed foods on the Epicollect5 Data Collection® platform.	Costa et al.55	2021	cross-sectional	To describe the Nova score for the consumption of ultra-processed foods (UPF) and evaluate its potential in reflecting the dietary share of UPF in Brazil.	13,7 minutes	
		Costa et al.56	2023		To describe two simple and easily derived diet quality scores and evaluate their performance in reflecting the dietary share of unprocessed or minimally processed whole plant foods and ultra-processed foods.	3 minutes	
**13**	CAAFE questionnaire (Food Consumption and Physical Activity Questionnaire for schoolchildren)	Costa57	2013	tool development	To develop a questionnaire for a web-based system for monitoring food intake and physical activity of schoolchildren attending 2nd to 5th grades	2 minutes	
		Costa et al.58	2013	tool development	To describe the usability evaluation of the Consumo Alimentar e Atividade Física de Escolares (CAAFE) questionnaire (Food Consumption and Physical Activity Questionnaire for schoolchildren), a new Web-based survey tool for the self-assessment of diet and physical activity by schoolchildren.		
		Davies et al.59	2015	validation	To assess the validity (matches, omissions and intrusions) and moderating factors of the CAAFE.		
**14**	Web dietary record	Dias60	2017	cross-sectional	To evaluate the association of usual food intake with iron *status* biomarkers among healthy women at childbearing age		
**15**	AMAMUNIC Project	Ducci61	2013	cross-sectional	To estimate the prevalence of breastfeeding and analyze the situation of the complementary feeding in children younger than one year in the city of Rolandia-Paraná.		
**16**	WhatsApp® Applied to Nutrition	Duque62	2020	tool development	WhatsApp® Applied to Nutrition: protocols for visual food recording using a messaging application.		
**17**	PDFG - Online version of the Previous Day Food Questionnaire for schoolchildren	Engel et al.63	2017	validation	To evaluate the validity of the web-based version of the Previous Day Food Questionnaire Online for schoolchildren from the 2nd to 5th grades of elementary school.		

Table S2. *Cont.*

Digital tool number	Digital tool	Author(Reference)	Year	Type of Study	Aims	Time (minutes)	Free Access
**18**	e-NutriHS - web-based	Folchetti et al.[65]	2015	tool development	To development of web-based online self-administered systems employing validated health related surveys.		
		Folchetti et al.[66]	2016	tool development	1) to analyze internationally available softwares addressed to the collection and analysis of data on nutrition; 2) to develop and implement the web system, e-NutriHS; 3) to develop the web system documentation; 4) to validate the data collected by NutriHS system; 5) to compare lifestyle habits (diet and physical activity), anthropometric measurements and biochemical profile of Nutrition undergraduates and nutritionists.	20-30 minutes	
**19**	NUTRISIM - System of health and nutrition monitoring - nutrition of school children	Galante[67]	2004	cross-sectional	To develop and validate a semi-quantitative food-frequency questionnaire on-line (QSFA) to evaluate the intake of Calcium and Iron.		
		Galante[68]	2007	validation	To develop and validate a self-administered computerized method to assess food consumption in adults using the internet		
		Galante[69]	2008	tool development	To develop an online semiquantitative food-frequency questionnaire for the evaluation of calcium and iron intake		
**20**	Quantitative Food Frequency Questionnaire (QUEFAC)	Hinnig[70]	2014	cross-sectional	To assess the reproducibility of QUEFAC for the sample as a whole and to evaluate the validity stratified by sex, age group and socioeconomic status.		
**21**	Eletronic Questionnaire – PeNSE	Ferreira64	2017	cross-sectional	To investigate the food consumption of students and its relation with individual, family, behavioral, and macroeconomic indicators.		
		IBGE - PeNSE, 2012[71]	2009		Support the monitoring of risk and health protection factors in schoolchildren in Brazil.		
		IBGE - PeNSE, 2012[72]	2013				
		IBGE - PeNSE, 2015[73]	2016				
		IBGE - PeNSE, 2019[74]	2021				
		Maia et al.[86]	2018		To identify and to analyze dietary patterns among Brazilian adolescents.		

Table S2. *Cont.*

Digital tool number	Digital tool	Author[(Reference)]	Year	Type of Study	Aims	Time (minutes)	Free Access
**22**	Digital questionnaire National Household Sample Survey (PNAD)	IBGE - PNAD, 2015[77]	2016	cross-sectional	To production of basic information for the study of the country's socioeconomic development.		
**23**	POF tool / Household Budget Survey tool	IBGE - POF 2017-2018[78]	2020	cross-sectional	To provide information on the composition of household budgets and the living conditions of the Brazilian population, including the subjective perception of quality of life, in addition to generating databases and studies on their nutritional profile.		
**24**	Web-CAAFE	Jesus, Assis, Kupek[79]	2017	validation	To evaluated the validity and reproducibility of the food consumption section of the questionnaire *Food Intake and Physical Activity of School Children* (Web-CAAFE), an Internet-based software for the qualitative measurement of food consumption by recalling the previous day.	13 minutes	
		Lobo et al.[83]	2019	cross-sectional	To identify dietary patterns (DPs) of children and adolescents participating in three cross-sectional surveys (2013–2015) and to test their associations with sociodemographic variables, physical activity (PAS), screen-based sedentary activity (SA), and weight status.	13 minutes	
**25**	Electronic Food Frequency Questionnaire	Karnopp[81]	2016	cross-sectional	To evaluate the consumption of ultraprocessados food and its relationship with serum lipid profile in adolescents 18 years of age.		
**26**	ENANI - The Brazilian National Survey on Child Nutrition -App24h	Kac G[80]	2020	cohort	To estimate and evaluate parameters related to breastfeeding practices and food consumption; anthropometric nutritional status and the epidemiology of micronutrient deficiencies.	15-20 minutes	
		Lacerda et al.[82]	2021	tool development	To describe methodological, operational and quality control aspects relating to the assessment of food consumption of children under 5 years old participating in the National Child Food and Nutrition Study (ENANI-2019).		
**27**	Electronic questionnaire on the Google Forms platform	Maciel[84]	2022	cross-sectional	To evaluate the incidence of the consumption of organic foods during the covid-19 pandemic.		

Table S2. *Cont.*

Digital tool number	Digital tool	Author(Reference)	Year	Type of Study	Aims	Time (minutes)	Free Access
**28**	Research Electronic Data Capture (REDCap)	Medeiros; Mainbourg[85]	2023	cross-sectional	To describe and analyze the food consumption of Amazonian riverside populations based on the food types consumed and reported by the families.		
**29**	Online questionnaire - Google Forms	Maynard et al.[88]	2020	cross-sectional	To analyze the eating pattern during the period of social distance as well as the anxiety caused by this new scenario.		
**30**	Online questionnaire - Google Forms	Meneghini, Netto[89]	2021	cross-sectional	To describe the nutritional and clinical profile of an adolescent with sickle cell disease, considering the consumption of macro and micronutrients, based on eating habits and anthropometric assessment.		
**31**	Nutrabem Application (Appn)	Mescoloto[90]	2018	validation	To Verify the validity of the Nutrabem Application as a tool for recording Food intake in adults.		
		Mescoloto, Caivano, Domene[91]	2017	cross-sectional	Evaluate the use of the Nutrabem (*São Paulo*, Brasil) mobile application as a tool for measurement of food intake among university students.		
**32**	The Food Frequency Questionnaire ELSA-Brasil	Molina et al.[92]	2013	tool development	Development of the Food Frequency Questionaire used in the Longitudinal Study of Adult Health-Brazil and analyze how diet exposes individuals to cardiovascular diseases and type 2 diabetes Mellitus.		
**33**	Web-based self-completed 24-h recall designed to categorise food intake according to Nova groups - Nova24h	Neri et al.[93]	2023	cohort	This paper describes the first web-based self-completed 24-h recall designed to categorise food intake according to Nova groups - Nova24h - and its agreement with a reference tool in estimating the dietary relative contribution of the four Nova food groups (% of total energy intake).	15 minutes	
**34**	Online semi-quantitative food frequency questionnaire (QSFA)	Ochsenhofer[95]	2007	validation	Validate an online semi-quantitative food frequency questionnaire (QSFA) to estimate iron and calcium intake in relation to 24-hour recalls (RH24's).		
**35**	Questionnaire QUACEB, (Illustrated Questionnaire on Food Consumption for Brazilian Schoolchildren).	Oliveira et al.[96]	2023	validation	To develop and validate the Illustrated Questionnaire on Food Consumption for Brazilian Schoolchildren (QUACEB) of 6 to 10 years old, which is a self-reported Illustrated recall.		

Table S2. *Cont.*

Digital tool number	Digital tool	Author(Reference)	Year	Type of Study	Aims	Time (minutes)	Free Access
**36**	GloboDiet	Steluti et al.[9]	2020	tool development	To develop a tool to evaluate food consumption, providing monitoring of the diet in the country and investigating the relationship between diet and health outcomes - The Brazilian version of the software GloboDiet.		
		Oliveira et al.[98]	2022	cross-sectional	To compare the identification of energy intake underreporters using different predictive equations and instruments to collect dietary data (Globodiet).		
**37**	Food Frequency Questionnaire Simplified for Adolescents (QFASA)	Ramos[99]	2013	cross-sectional	Applying the Simplified Food Frequency Questionnaire for Adolescents (QFASA) in digital environment to evaluation of usual diet of school children in Salesópolis - SP.	20-30 minutes	
		Voci[114]	2011	cross-sectional	To verify possible associations between food insecurity and food patterns, participation in social programs and other factors among adolescents enrolled in public schools of Piracicaba (SP).		
**38**	PENSSAN network application	I National Survey on Food Insecurity in the Context of the Covid-19 Pandemic in Brazil[100]	2021	cross-sectional	To Monitor Food Security and levels of Food Insecurity in the homes of Brazilian families, in the face of the economic and health crisis scenario, resulting from the COVID-19 pandemic, aiming to provide quick and reliable information, capable of influencing the decision-making process of public actions and the mobilization of organized civil society.		
		II National Survey on Food Insecurity in the Context of the Covid-19 Pandemic in Brazil[101]	2022				

Table S2. *Cont.*

Digital tool number	Digital tool	Author(Reference)	Year	Type of Study	Aims	Time (minutes)	Free Access
**39**	NUTRISIM - System of health and nutrition monitoring - nutrition of school children"	Ruggeri et al.[103]	2012	tool development	To develop a structured and automated 24-hour Dietary Recall (R24ec) to evaluate schoolchildren and adolescent food intake, with both online and offline modes.		
		Ruggeri et al.[104]	2013	cross-sectional	To evaluate the usability of the "System of health and nutrition monitoring - nutrition of school children" (NUTRISIM).		
**40**	Online Questionnaire - Google Forms	Ruiz-Roso et al.[105]	2020	cross-sectional	To study nutritional modifications during COVID-19 confinement in adolescents aged 10 to 19 years, compare them with their usual diet and dietary guidelines, and identify variables that may have influenced changes.		
**41**	NutriCogni	Santos et al.106	2019	tool development	To describe the development and evaluation of mobile app prototype on behavioral strategies based on Intuitive Eating assumptions to assist nutritionists in identifying dietary patterns.		
**42**	Tool for assessing the consumption of ultra-processed foods.	Sattamini[107]	2019	validation	Development, adaptation and validation of tools to assess two dimensions of diet quality in Brazil. Methods - Adaptation of the Minimum Dietary Diversity for Women (MDD-W) indicator of the Food and Agriculture Organization (FAO).	15 minutes	
**43**	Semiquantitative, digital and self-administered FFQ	Schneider et al.[8]	2016	tool development	To describe the development of a digital and self-reported food frequency questionnaire (FFQ), created to the 1982 and 1993 Pelotas Birth Cohorts.	12 minutes	
**44**	EANSE FORMS	Silva et al.[109]	2021	cross-sectional	To describe the methodological and prototyping process of the digital form for studies on food, nutrition, health and aging: Eanse Forms.		
**45**	Electronic questionnaire of markers of food consumption adapted from the National System of Food and Nutritional Surveillance (SISVAN).	Silva et al.[110]	2021	cross-sectional	To analyze the nutritional status and food consumption of university Teachers from a private institution in the municipality of Várzea Grande-MT.		

Table S2. *Cont.*

Digital tool number	Digital tool	Author(Reference)	Year	Type of Study	Aims	Time (minutes)	Free Access
**46**	Diet quality was assessed with ESQUADA using the Epicollect5 application.	Silva et al.[111]	2021	tool development	To analyze the association between diet quality assessed with the Diet Quality Scale (ESQUADA) and the nutritional status and metabolic risk in adults.		
**47**	NutriNet Brasil	Steele et al.[113]	2020	cohort	To describe the dietary characteristics of participants in the NutriNet Brasil cohort immediately before and during the covid-19 pandemic.		
**48**	FODMAPs ("Fermentable Oligosaccharides, Disaccharides, Monosaccharides and Polyols") - FODMAP Project	Yamashita et al.[115]	2019	validation	To develop and validate a short food frequency questionnaire (FFQ) to assess FODMAP usual consumption in adults with irritable bowel syndrome (IBS), along with a photo album of food portion sizes		

Table S3. Summary of characteristics for validation studies (n = 11) included in the scoping review.

Validation study number	Digital tool	Author(Reference)	Year	Validation Process	
				Reference/"Good Standard" Method	Main results
**1**	CUME – Online questionnaire – The Cohort of Universities of Minas Gerais	Azarias[27]	2020	Multiple pass 24HR technique.	The CUME project's online and self-completed FFQ has satisfactory validity and reproducibility and can be used to analyze the association between food consumption and chronic non-communicable diseases in adults with a high level of education.
**2**	Digital Food Guide (DQI-DFG) – for a second version.	Caivano; Colugnati, Domene[50]	2019	24-hour recall (24HR)	The DQI-DFG showed 18consistent validity and reliability characteristics according to the criteria adopted in this study.
**3**	CAAFE questionnaire (Food Consumption and Physical Activity Questionnaire for schoolchildren)	Davies et al.[59]	2015	Direct observation/Dietary diary	The CAAFE questionnaire provided a reasonable, although further studies are required to improve its validity.
**4**	PDFG – Online version of the Previous Day Food Questionnaire for schoolchildren	Engel et al.[63]	2017	Direct observation	The PDFQ Online possessed satisfactory validity for the evaluation of dietary intake among school children 7 to 12 years old.
**5**	NUTRISIM - System of health and nutrition monitoring - nutrition of school children	Galante[68]	2007	Questionnaire for System Usability	The study showed that the system is easy to learn and use, but the answers are scattered. The instrument proved to be a useful tool to monitor and evaluate health and dietary intake in epidemiologic studies.
**6**	Web-CAAFE	Jesus, Assis, Kupek[79]	2017	Direct observation	The Web-CAAFE was a valid and reliable instrument for the evaluation of food consumption when applied to students in grades 2 to 5 of public schools.

Table S3. *Cont.*

Validation study number	Digital tool	Author[(Reference)]	Year	Validation Process	
**7**	Nutrabem application (appN)	Mescoloto[90]	2018	24-hour recall (24HR)	The appN's validation study can be considered robust due to the methodological protocol adopted. The results allow to indicate the potential of appN as a tool for recording dietary intake due to strong correlations compared to the reference instrument. According to users' perception, the application indicated good usability.
**8**	Online semi-quantitative food frequency questionnaire (QSFA)	Ochsenhofer[95]	2007	24-hour recall (24HR)	The results indicated that the QSFA online was validates for iron and calcium.
**9**	Questionnaire was given the acronym QUACEB, corresponding to the initials of the name in Portuguese: "Questionário de Consumo Alimentar para Crianças Escolares Brasileiras" (Illustrated Questionnaire on Food Consumption for Brazilian Schoolchildren).	Oliveira et al.[96]	2023	24-hour recall (24HR)	The food consumption questionnaire is valid for elementary schoolchildren of 6 to 10 years old and can be applied in research to assess the dietary patterns of children in Brazil.
**10**	Tool for assessing the consumption of ultra-processed foods.	Sattamini[107]	2019	24-hour recall (24HR)	The simplified instruments of dietary diversity and ultra-processed food consumption presented satisfactory validity. These instruments can be useful for research on diet quality in different monitoring contexts and population diagnoses, as well as for intervention studies and designing of public health policies in Brazil.
**11**	FODMAPs ("Fermentable Oligosaccharides, Disaccharides, Monosaccharides and Polyols") - FODMAP Project	Yamashita et al.[115]	2019	24-hour recall (24HR)	The instrument presents good reproducibility for all groups of FODMAPs, good validity for lactose and weaker validity for fructose, polyols and oligosaccharides.

File S1. PRISMA-ScR (Preferred Reporting Items for Systematic Reviews and Meta-Analyses for Scoping Reviews) guidelines.

Preferred Reporting Items for Systematic reviews and Meta-Analyses extension for Scoping Reviews (PRISMA-ScR) Checklist

SECTION	ITEM	PRISMA-ScR CHECKLIST ITEM	REPORTED ON PAGE #
**TITLE**			
Title	1	Identify the report as a scoping review.	1
**ABSTRACT**			
Structured summary	2	Provide a structured summary that includes (as applicable): background, objectives, eligibility criteria, sources of evidence, charting methods, results, and conclusions that relate to the review questions and objectives.	Not applicable
**INTRODUCTION**			
Rationale	3	Describe the rationale for the review in the context of what is already known. Explain why the review questions/objectives lend themselves to a scoping review approach.	2
Objectives	4	Provide an explicit statement of the questions and objectives being addressed with reference to their key elements (e.g., population or participants, concepts, and context) or other relevant key elements used to conceptualize the review questions and/or objectives.	2
**METHODS**			
Protocol and registration	5	Indicate whether a review protocol exists; state if and where it can be accessed (e.g., a Web address); and if available, provide registration information, including the registration number.	*The review protocol and registration to be included under methods. As the international prospective register of systematic reviews (PROSPERO) does not accept the registration of scoping reviews, a registration number for this scoping review is not available.*
Eligibility criteria	6	Specify characteristics of the sources of evidence used as eligibility criteria (e.g., years considered, language, and publication status), and provide a rationale.	2
Information sources*	7	Describe all information sources in the search (e.g., databases with dates of coverage and contact with authors to identify additional sources), as well as the date the most recent search was executed.	2-3

SECTION	ITEM	PRISMA-ScR CHECKLIST ITEM	REPORTED ON PAGE #
Search	8	Present the full electronic search strategy for at least 1 database, including any limits used, such that it could be repeated.	3 and Supplementary File S2
Selection of sources of evidence†	9	State the process for selecting sources of evidence (i.e., screening and eligibility) included in the scoping review.	4
Data charting process‡	10	Describe the methods of charting data from the included sources of evidence (e.g., calibrated forms or forms that have been tested by the team before their use, and whether data charting was done independently or in duplicate) and any processes for obtaining and confirming data from investigators.	3
Data items	11	List and define all variables for which data were sought and any assumptions and simplifications made.	3
Critical appraisal of individual sources of evidence§	12	If done, provide a rationale for conducting a critical appraisal of included sources of evidence; describe the methods used and how this information was used in any data synthesis (if appropriate).	Not applicable
Synthesis of results	13	Describe the methods of handling and summarizing the data that were charted.	Supplementary Table S1 and S2 Pages 4-7
**RESULTS**			
Selection of sources of evidence	14	Give numbers of sources of evidence screened, assessed for eligibility, and included in the review, with reasons for exclusions at each stage, ideally using a flow diagram.	4
Characteristics of sources of evidence	15	For each source of evidence, present characteristics for which data were charted and provide the citations.	Pages 3-7 Supplementary Table S1 and S2
Critical appraisal within sources of evidence	16	If done, present data on critical appraisal of included sources of evidence (see item 12).	Not applicable
Results of individual sources of evidence	17	For each included source of evidence, present the relevant data that were charted that relate to the review questions and objectives.	Pages 3-7 Supplementary Table S1 and S2
Synthesis of results	18	Summarize and/or present the charting results as they relate to the review questions and objectives.	Pages 3-7 Supplementary Table S1 and S2
**DISCUSSION**			
Summary of evidence	19	Summarize the main results (including an overview of concepts, themes, and types of evidence available), link to the review questions and objectives, and consider the relevance to key groups.	7-11
Limitations	20	Discuss the limitations of the scoping review process.	9-10
Conclusions	21	Provide a general interpretation of the results with respect to the review questions and objectives, as well as potential implications and/or next steps.	10

SECTION	ITEM	PRISMA-ScR CHECKLIST ITEM	REPORTED ON PAGE #
**FUNDING**			
Funding	22	Describe sources of funding for the included sources of evidence, as well as sources of funding for the scoping review. Describe the role of the funders of the scoping review.	Not applicable

JBI = Joanna Briggs Institute; PRISMA-ScR = Preferred Reporting Items for Systematic reviews and Meta-Analyses extension for Scoping Reviews.
* Where *sources of evidence* (see second footnote) are compiled from, such as bibliographic databases, social media platforms, and Web sites.
† A more inclusive/heterogeneous term used to account for the different types of evidence or data sources (e.g., quantitative and/or qualitative research, expert opinion, and policy documents) that may be eligible in a scoping review as opposed to only studies. This is not to be confused with *information sources* (see first footnote).
‡ The frameworks by Arksey and O'Malley (6) and Levac and colleagues (7) and the JBI guidance (4, 5) refer to the process of data extraction in a scoping review as data charting.
§ The process of systematically examining research evidence to assess its validity, results, and relevance before using it to inform a decision. This term is used for items 12 and 19 instead of "risk of bias" (which is more applicable to systematic reviews of interventions) to include and acknowledge the various sources of evidence that may be used in a scoping review (e.g., quantitative and/or qualitative research, expert opinion, and policy document).

From: Tricco AC, Lillie E, Zarin W, O'Brien KK, Colquhoun H, Levac D, et al. PRISMA Extension for Scoping Reviews (PRISMAScR): Checklist and Explanation. Ann Intern Med. 2018;169:467–473. doi: 10.7326/M18-0850.

File S2 – The detailed search in the databases

Database	Search strategies
Pubmed	(Adults* [mh] OR adults[tiab] OR child*[mh] OR child*[tiab]OR Aged[mh] OR aged[tiab] OR elderly[tiab] OR adolescent*[mh] OR adolescente*[tiab] OR teen[tiab]*) AND ("Surveys and questionnaires" [mh] OR survey*[tiab] OR design[tiab] OR methods [tiab]OR methodology[tiab] OR community [tiab]OR baseline[tiab] OR respondent*[tiab] OR technique* OR questionnaire* OR nonresppondent*) AND ("electronic health records"[mh]OR electronic[tiab] OR computerized[tiab] OR health[tiab] OR medical[tiab]OR record*[tiab]) AND ("medical informatics applications"[mh] OR medical[tiab] OR Informatic*[tiab] OR application*[tiab]) AND (software [mh] OR computer[tiab] OR technology*[tiab] OR program*[tiab] OR tool*[tiab] OR "computer applications software" [tiab] OR programming[tiab]) AND ("mobile applications"[mh] OR app[tiab] OR "portable electronic apps" [tiab] OR "Portable software"[tiab]) AND ("Diet records"[mh] OR diet[tiab] OR food[tiab] OR dietary[tiab] OR record [tiab] OR diaries[tiab] OR diary[tiab] OR dietary[tiab]) AND ("nutrition surveys"[mh] OR nutrition[tiab] OR nutritional[tiab] OR "nutrition examination"[tiab] OR "national health"[tiab] OR (survey*[tiab]) AND ("Diet surveys"[mh]) AND (brazil[mh] OR "south america"[tiab]) AND ("Nutrition assessment"[mh] OR nutrition[tiab] OR nutritional[tiab] OR assessment*[tiab] OR indexes[tiab] OR indice*[tiab]) AND ("health surveys"[mh] OR health[tiab] OR survey*[tiab]) AND (eating[mh] OR eating[tiab] OR food[tiab] OR intake[tiab] OR ingestion[tiab])
Lilacs	(tw:(mh:"Consumo de Alimentos" OR (Food Consumption) OR (Consumo de Alimentos) OR (Consumo de Alimentos) OR (Consumo Alimentar) OR mh:SP6.056.237$)) AND (tw:(mh:Brasil OR (Brazil) OR (Brasil) OR (Brasil) OR hh:Z01.107.757.176$)) AND (tw:(mh:"Inquéritos Nutricionais" OR (Nutrition Surveys) OR (Encuestas Nutricionales) OR (Inquéritos Nutricionais) OR (Levantamentos Nacionais de Saúde e Nutrição) OR (Levantamentos Nutricionais) OR (Monitoramento do Estado Nutricional) OR (NHANES) OR (National Health and Nutrition Examination Survey) OR mh:E05.318.308.980.485$ OR mh:N05.715.360.300.800.469$ OR mh:N06.850.505.616$ OR mh:N06.850.520.308.980.469$ OR mh:SP6.051.222.158$)) AND (tw:(mh:Software OR (Software) OR (Programas Informáticos) OR (Software) OR (Engenharia de Software) OR (Ferramentas de Software) OR (Programas de Computador e Programação) OR (Programas de Computação) OR (Programas para Computadores) OR (Programação e Programas de Computador) OR (Software Proprietário) OR (Software de Aplicativos) OR (Software de Aplicativos de Computador) OR (Software de Aplicações) OR (Software de Aplicações Informáticas) OR (Suporte Lógico de um Sistema Informático) OR mh:L01.224.900$))
Embase	('dietary intake'/exp OR 'dietary intake' OR (dietary AND intake)) AND ('food intake'/exp OR 'food intake' OR (('food'/exp OR food) AND intake)) AND ('questionnaire'/exp OR questionnaire) AND ('brazil'/exp OR brazil) AND ('software'/exp OR software)
Web Of Science	(TS=(“diet Surveys” OR “diet Survey” OR survey OR diet OR surveys)) AND (Ts=(“nutrition Surveys” OR “nutrition Survey” OR “nutritional survey” OR “nutritional surveys” OR survey OR nutrition OR Survey OR “nutritional surveys” OR nutritional)) AND (Ts=(“Food Consumption”)) AND (Ts=(Eating OR “food Intake” OR ingestion OR intake OR food)) AND (Ts=(Software OR “computer applications” OR software OR “computer programs” OR “computer programs and programming” OR “computer software” OR “computer software applications” OR “software tools”)) AND (Ts=(Brazil*))
Scopus	(TITLE-ABS-KEY ("nutrition surveys") AND TITLE-ABS-KEY (brazil) AND TITLE-ABS-KEY (software))

Science Direct	("nutrition surveys" AND (brazil OR brazilian) AND (software) AND (intake) AND (recall))
Journals@Ovid Full Text (Ovid)	BRAZILIAN DIETARY INTAKE DIGITAL TOOLS SURVEYS (pesquisa básica)
Free Medical Journal (The Journal of Nutrition, Plos Medicine)	(nutrition surveys AND brazil AND software AND brazilian)<br><br>(((((everything:"nutrition surveys") AND everything:brazil) OR everything:brazilian) AND everything:"food intake") AND everything:technology) AND everything:software
Crossref	("consumo alimentar") AND ("inquéritos nutricionais" OR "inquéritos epidemiológicos") AND questionários AND Brasil AND (softwares) AND (tecnologia) AND (questionários)
Banco de Teses e Dissertações	("inquéritos nutricionais") AND (brasil)
Biblioteca Digital de Teses e Dissertações	"inquéritos nutricionais"<br>"Questionário de frequência alimentar"
Google Scholar	("consumo alimentar") AND ("inquéritos nutricionais" OR "inquéritos epidemiológicos") AND questionários AND Brasil AND (softwares) AND (tecnologia)
